# Preparation of Smart Surfaces Based on PNaSS@PEDOT Microspheres: Testing of *E. coli* Detection

**DOI:** 10.3390/s22072784

**Published:** 2022-04-05

**Authors:** Elena Tomšík, Svetlana Laishevkina, Jan Svoboda, Kristýna Gunar, Jiřina Hromádková, Natalia Shevchenko

**Affiliations:** 1 Institute of Macromolecular Chemistry, Academy of Sciences of the Czech Republic, Heyrovsky Sq. 2, 162 06 Prague, Czech Republic; s.laishevkina@gmail.com (S.L.); svoboda@imc.cas.cz (J.S.); gunar@imc.cas.cz (K.G.); hromadkova@imc.cas.cz (J.H.); 2 Institute of Macromolecular Compounds, Russian Academy of Sciences, Bolshoy pr., 31, 199004 Saint-Petersburg, Russia

**Keywords:** PEDOT, PNaSS, impedance, smart surface, microspheres

## Abstract

The main task of the research is to acquire fundamental knowledge about the effect of polymer structure on the physicochemical properties of films. A novel meta-material that can be used in manufacturing sensor layers was developed as a model. At the first stage, poly(sodium 4-styrenesulfonate) (**PNaSS**) cross-linked microspheres are synthesized (which are based on strong polyelectrolytes containing sulfo groups in each monomer unit), and at the second stage, **PNaSS@PEDOT** microspheres are formed. The poly(3,4-ethylenedioxythiophene) (**PEDOT**) shell was obtained by the acid-assisted self-polymerization of the monomer; this process is biologically safe and thus suitable for biomedical applications. The suitability of electrochemical impedance spectroscopy for *E. coli* detection was tested; it was revealed that the attached bacterial wall was destroyed upon application of constant oxidation potential (higher than 0.5 V), which makes the **PNaSS@PEDOT** microsphere particles promising materials for the development of antifouling coatings. Furthermore, under open-circuit conditions, the walls of *E. coli* bacteria were not destroyed, which opens up the possibility of employing such meta-materials as sensor films. Scanning electron microscopy, X-ray photoelectron spectroscopy, water contact angle, and wide-angle X-ray diffraction methods were applied in order to characterize the **PNaSS@PEDOT** films.

## 1. Introduction

New strategies for the fabrication of meta-materials are of great importance in the development of modern technology and fundamental science. Despite the tremendous progress that has already been made in understanding the synthesis of poly(3,4-ethylenedioxythiophene)-polystyrene sulfonate (PEDOT-PSS) composites, there are some unresolved problems. It is still unclear how to create three-dimensionally ordered structures based on such composites, how to increase charge density in the composites, and how to effectively remove reaction products so that the resulting meta-material is considered eco-friendly [1]. Usually, the preparation of 3D meta-materials involves the use of labor-intensive and expensive equipment. This problem can be solved by using particles capable of self-assembling into hierarchical structures. The development of methods for obtaining such hierarchical meta-materials based on PEDOT/PSS structures (which can act as a working electrode in electrochemical impedance spectroscopy) offers possibilities of creating new smart materials: biosensors, antifouling coatings, etc. [2,3,4,5,6].

The semiconducting material is a key component of electrochemical biosensors [7,8]. Although there are examples of the successful application of inorganic materials in electrical biosensors [9], the use of organic semiconducting materials holds greater promise [10,11,12,13]. In addition to electrical conductivity, organic semiconductors demonstrate the properties typical of plastics, such as solubility, mechanical strength, and adjustable physicochemical characteristics [14]. A large variety of electroconductive polymers and their derivatives find application as biosensors [15,16,17,18]. The examples of well-known and thoroughly studied polymer semiconductors include polypyrrole (PPy), polyaniline (PANI), and polythiophenes. However, the most common organic conductor is poly(3,4-ethylenedioxythiophene) (PEDOT); it is frequently used in the doped form stabilized with polystyrene sulfonate) (PSS) polyanion [19,20]. 

Impedance sensors with the working electrodes including **PEDOT** as an active material are used for the detection of different substances [21,22,23,24,25,26,27,28,29]. The key parameter that allows **PEDOT** to be applied in sensors is a high-quality interface between biological tissue and electronics [30]. 

**Scheme 1:**
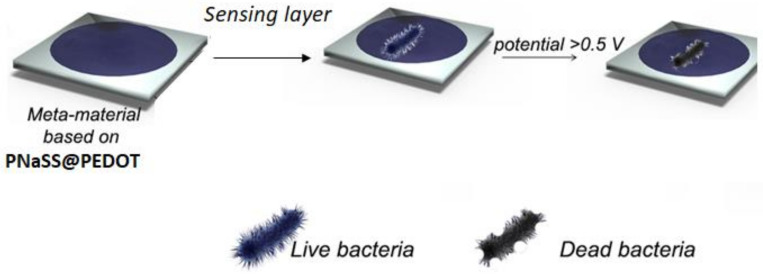
Schema of experimental concept for creation of meta-materials based on **PNaSS@PEDOT** microspheres.

The adhesion of bacteria on surfaces can lead to serious problems (e.g., contamination of implants, failure of medical devices, and/or threats to public health). Preparation and studies of antibacterial films attract an increasing interest of researchers due to possible biomedical applications (Scheme 1) [31,32,33,34,35,36,37,38,39,40,41,42].

The main strategy that could be used to design biosensors for the detection of bacteria is the development of meta-materials as an antifouling coating (Scheme 1), which could be further regenerated by applying constant potential. Such a sensor is studied in detail in the current paper.

In this paper, the method of preparation of **PNaSS@PEDOT** microsphere particles is developed. We demonstrate the fundamental possibility of forming microspheres based on **PNaSS** by the method of inverse emulsion polymerization, and the ability to form a composite with PEDOT. The advantage of applying **PNaSS** cross-linked microspheres is the presence of sulfo groups in each monomer unit [43]. The sensing layer obtained from **PNaSS@PEDOT** microspheres is stable and, more importantly, reproducible. Moreover, the new acid-assisted self-polymerization method for EDOT monomer is biologically friendly, and the final **PEDOT** shell is composed of the polymer in the reduced state [44,45], which makes the **PNaSS@PEDOT** microspheres suitable as an antifouling coating layer, as shown in Scheme 1. On the one hand, this structure provides limited affinity of the surface towards bacteria; on the other hand, the reduced state facilitates internal conductivity by the presence of the core containing the sulfonate groups in each monomer unit.

The electrochemical impedance spectroscopy, stability, antifouling properties against proteins, and antimicrobial properties against *Escherichia (E.) coli* bacteria were systematically investigated.

## 2. Materials and Methods

### 2.1. Materials

Cross-linked microspheres were synthesized according to the SI (see Supplementary Table S1), and were used for further preparation of **PNaSS@PEDOT** microspheres. The monomer (3,4-ethylene dioxythiophene—EDOT) was bought from Sigma-Aldrich and was used without further purification. Formic acid 98% GR and ethyl alcohol 99.8% for UV spectroscopy were bought from Lach-ner, Czech Republic, and were used as received.

### 2.2. Preparation of **PNaSS@PEDOT** Microspheres Composite

Cross-linked microspheres were added to 4.7 mL of concentrated formic acid; 56 µL of EDOT monomer was dissolved in 2 mL of ethyl alcohol and then added to the solution of cross-linked microspheres. The reaction of acid-assisted self-polymerization occurred and a stable solution of composite material **PNaSS@PEDOT** microspheres in formic acid was obtained. The film **PNaSS@PEDOT** microspheres were deposited using the doctor blade technique on fluorine-doped tin oxide (FTO) glasses.

### 2.3. Bacterial Adhesion on **PEDOT** Films

Bacteria Escherichia (*E.*) *coli* BL21 (kindly provided by Dr. Karel Holada, First Faculty of Medicine, Charles University, Prague, Czech Republic) were incubated in liquid Luria Broth (LB, Sigma-Aldrich) at 37 °C. An overnight culture of *E. coli* was resuspended in Hank’s balanced salt solution (0.14 M NaCl, 5.4 mM KCl, 0.44 mM KH_2_PO_4_, 5.5 mM glucose, 0.34 mM Na_2_HPO_4_, 4.2 mM NaHCO_3_) and diluted in LB to a concentration of 10^6^ colony forming units (CFU)/mL. FTO slides coated with **PNaSS@PEDOT** microsphere films were placed into a 24-well plate (TPP, Switzerland), and 3 mL of LB media with *E. coli* was added. The plates were incubated for 4 h at 37 °C without shaking; afterwards, the LB medium was discarded and glass slides were washed in HBSS. The slides were stored submerged in HBSS until further testing.

Ten electrodes were used in order to check the reproducibility of the measured results.

### 2.4. **PNaSS@PEDOT** Microspheres Film Characterization

Electrochemical impedance spectroscopy (EIS) was performed in the frequency range from 10 kHz to 0.1 Hz at open circuit potential (OCP). The Kroning–Kramers test was applied to verify the obtained EIS data. 

X-ray photoelectron spectroscopy (XPS) measurements were carried out with a K-Alpha + spectrometer (ThermoFisher Scientific, East Grinstead, UK). The samples were analyzed using a micro-focused, monochromated Al Kα X-ray source (400 µm spot size) at an angle of incidence of 30° (measured from the surface) and an emission angle normal to the surface. The kinetic energy of the electrons was measured using a 180° hemispherical energy analyzer operated in the constant analyzer energy mode (CAE) at 200 eV and 50 eV pass energy for the survey and high-resolution spectra, respectively. Data acquisition and processing were performed using Thermo Advantage software. The XPS spectra were fitted with Voigt profiles obtained by convolving Lorentzian and Gaussian functions. The analyzer transmission function, Scofield sensitivity factors, and effective attenuation lengths (EALs) for photoelectrons were applied for quantification. The EALs were calculated using the standard TPP-2M formalism. All spectra were referenced to the C1s peak of hydrocarbons at 285.0 eV. The BE scale was controlled by the well-known position of the photoelectron C-C and C-H, C-O and C(=O)-O C1s peaks of polyethylene terephthalate and Cu 2p, Ag 3d, and Au 4f peaks of metallic Cu, Ag and Au, respectively. The BE uncertainty of the reported measurements and analysis is in the range of ±0.1 eV.

The water contact angles (WCA) were measured with a OCA20 (DataPhysics Instruments, Filderstadt, Germany), and the static CA was determined after 10 s of applying a water droplet of 5 µL on the polymer film. A minimum of five drops were measured.

The surface morphology was analyzed by scanning electron microscopy (SEM). The scanning electron micrographs were obtained using a JEOL 6400 microscope. The transmission electron micrographs were obtained using a Tecnai G2 Spirit (FEI Czech Republic s.r.o., Prague, Czech Republic).

Wide-angle X-ray diffraction (XRD) patterns were obtained using a high-resolution diffractometer Explorer (GNR Analytical Instruments, Novara, Italy). The instrument is equipped with a one-dimensional silicon strip detector, Mythen 1K (DECTRIS AG, Baden-Daettwil, Switzerland). The samples were measured in reflection mode. The radiation CuKα (wavelength λ = 1.54 Å) monochromatized with Ni foil (β filter) was used for diffraction. The measurement was done in the range 2θ = 2–25° with step 0.1°. The exposure time at each step was 10 s.

The specific surface area and pore size distribution of the microspheres synthesized were assessed by a nitrogen gas sorption analyzer (NOVA 1200, Quantachrome GmbH & Co. KG, Odelzhausen, Germany), and were determined by a Multipoint BET (Brunauer–Emmett–Teller) method. The DFT (Density Functional Theory) method was applied to calculate the pore size distribution from the analysis of the desorption branches of the isotherms. Preliminarily, water was removed from the microsphere dispersions by evaporation at a temperature of 40 °C under reduced pressure using a “Laborota4011” rotary evaporator. The sample was degassed before the measurements by nitrogen flow under reduced pressure. 

## 3. Results and Discussion

### 3.1. **PNaSS@PEDOT** Microspheres Synthesis and Film Deposition

The synergetic properties of 3,4-ethylenedioxythiophene polymer and conjugated polyelectrolytes make them innovative and promising materials for creating biological interfaces, particularly sensing layers in biosensors. 

**Scheme 2:**
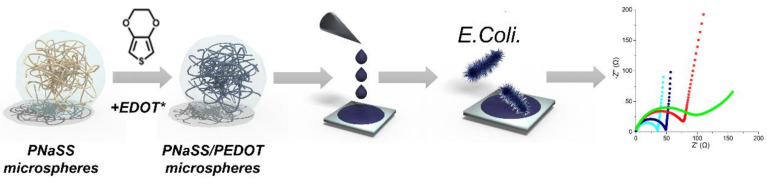
Scheme of the synthetic way to prepare **PNaSS@PEDOT** microspheres, the way of its deposition on FTO surface, further interaction with bacteria *E. coli*, and electrochemical impedance spectroscopy is the method of choice.

Recently, an innovative synthetic way for microspheres with a high concentration of sulfo groups on their surface has been developed [43]. The general procedure for the synthesis of microspheres is described in detail in SI (see also Figure S1). The surface charge density of -SO_3_ groups in these microspheres was 1.54 × 10^−4^ eq/g. The microspheres were used as cores in the preparation of the **PNaSS@PEDOT** microsphere particles (Scheme 2). The polymer film was obtained in the process of acid-assisted self-polymerization of the monomer (3,4-ethylenedioxythiophene, EDOT); the reaction was first described by our research group [44,45]. The synthesis can be summarized as follows: the prepared microspheres were dispersed in concentrated formic acid, and EDOT monomer was added to the dispersion. The deposition of **PEDOT** started immediately, and the uniform **PEDOT** layer was formed on the surface of the microspheres (Scheme 2). 

**Figure 1:**
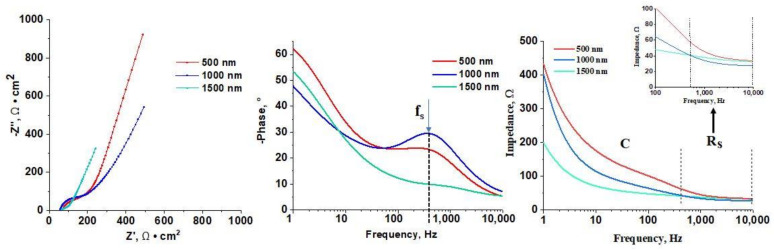
Nyquist plots of **PNaSS@PEDOT** microsphere films with different thickness (**a**), corresponding Bode plots (**b**), and impedance vs. frequency (**c**); the inset is the frequencies range from 500 to 10 kHz.

This synthetic method is suitable for applications in biosensor development, because no by-products are formed during **PEDOT** synthesis, and thus, the suspension does not need purification. The suspension of **PNaSS@PEDOT** microspheres particles is stable for at least 6 months and can be deposited onto any desirable surface to form a sensing layer. In our research, fluorine-doped tin oxide (FTO) glasses were used as electrode supports onto which a sensing layer was deposited. The doctor blade method was found to be the optimal technique for the deposition of stable and reproducible films (for details see SI, Figure S2). The drop-casting and spin-coating methods were tested as well, but the parameters of the resulting films (reproducibility, thickness, and stability) turned out to be unsuitable for our purposes. Our next step was to find the optimal thickness of the sensor film. The thickness of the films varied from 500 to 1500 nm, and the results of electrochemical impedance spectroscopy (EIS) measurements in the form of the Nyquist plots, Bode plots and impedance vs. frequency plots are presented in Figure 1. For comparison, the EIS of pure FTO electrode in PBS solution is presented in the Supporting Information (Figure S3).

According to the obtained data, the optimal thickness of the **PNaSS@PEDOT** microspheres layer is 1000 nm. When the data are considered from a complementary perspective, the resistive nature of the system is clear through the zero phase φ value at high frequencies from 10 kHz to 500 Hz (Figure 1b, Bode plot). In this frequency range, the impedance does not depend on frequency (Figure 1c). According to D.C. Martin et al., the characteristic frequency fc is the frequency at which impedance goes from the low frequency dependent to the high frequency independent region [4]. Moreover, in this frequency range (from 10 kHz to 500 Hz), the impedance is only represented by electrolyte resistance Rs. On the other hand, at a low frequency, the impedance can be modeled by the capacitance of the film C. The concentration dependence of the impedance measured at different salt concentrations (NaCl, from 0.1 to 0.005 M) is presented in the Supporting Information (Figure S4). The resulting impedance values are virtually similar at low frequencies (-Z″ does not change), but at high frequencies, real parts of the impedance (Z′) differ; thus, the impedance decreases with increasing salt concentration, which corresponds to a decrease in electrolyte resistance R_s_.

The detailed impedance of **PNaSS@PEDOT** microspheres films is presented in Figure 1c (inset); it is obvious that the lowest impedance was recorded for the film at 1000 nm thick. That is why the further experiments involved films of this thickness.

### 3.2. Structure and Surface Characterization

The acid-assisted self-polymerization of the EDOT monomer without the formation of by-products was first revealed by our research group and studied in detail [44]. The analysis of high-resolution core-level S2p X-ray photoelectron spectra (XPS) confirmed that only polymers with reduced monomer units were formed during acid-assisted self-polymerization [44].

**Figure 2:**
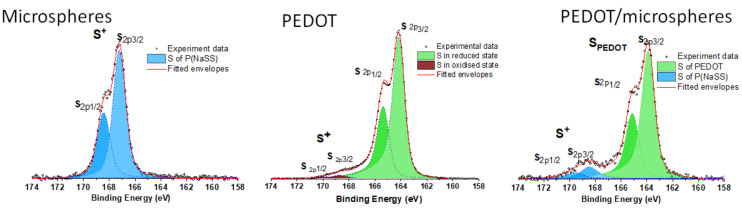
High-resolution core-level S2p X-ray photoelectron spectra of core microspheres, **PEDOT** and **PNaSS@PEDOT** microspheres.

The chemical structure of the **PNaSS@PEDOT** microspheres was confirmed by high-resolution core-level S2p XPS (Figure 2). XPS was first applied in the studies of microspheres; only a spin-orbit split peak at ~168 eV (corresponding to sulfur in the oxidized state) was observed. On the other hand, **PEDOT** has a peak at ~164 eV, which corresponds to the reduced sulfur atom in thiophene fragment. A small shoulder at ~168 eV was recorded for **PEDOT** (~6%); our previous studies have demonstrated that this peak corresponded to thiophene sulfur that lost electron density due to the formation of hydrogen bonds between the formic acid and thiophene unit [44]. The final **PNaSS@PEDOT** microsphere material shows both the peaks corresponding to reduced thiophene sulfur (~84 atomic%, the values were calculated from the intensities of both peaks) and the peak attributed to oxidized sulfur (~16%) from the sulfo groups of microspheres and/or the thiophene sulfur that has diminished electron density due to the formation of hydrogen bonds between the formic acid and thiophene rings (Figure 2). The presence of sulfo groups in **PNaSS@PEDOT** microspheres plays an important role in the charge propagation in the sensing layer. This phenomenon was thoroughly studied by electrochemical impedance spectroscopy (EIS).

**Figure 3:**
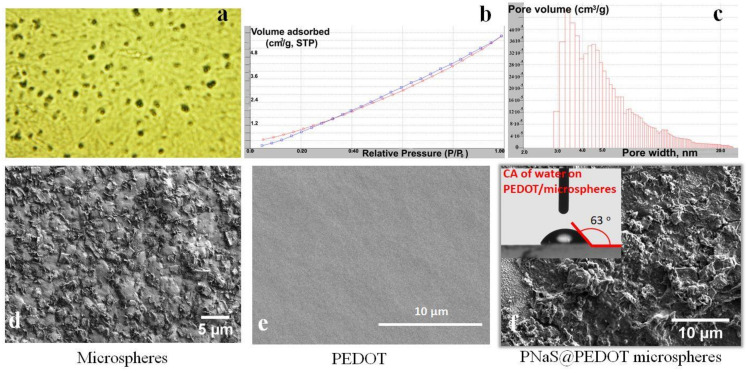
(**a**) Optical microscopy of **PNaSS** microspheres, (**b**) nitrogen adsorption–desorption isotherms of **PNaSS** microspheres, and (**c**) pore size distribution of **PNaSS** microspheres. (**d**) Scanning electron microscopy images of microspheres, (**e**) PEDOT, and (**f**) **PNaSS@PEDOT** microspheres film, and water contact angle (CA) of **PNaSS@PEDOT** microspheres.

It has been established by various research groups that the structure of **PEDOT** can be tuned through synthetic chemistry, and the physicochemical and/or electrochemical properties of the material can be controlled using a variety of strategies [46]. In our study, the microspheres of **PNaSS** ~0.5–5 µm in size were used (Figure 3a) [43]. The application of the method of inverse emulsion polymerization (which we used) makes it possible to obtain microspheres with only a wide size distribution, since there is no unified theory that would describe the regularities of inverse emulsion polymerization, and research in this direction is still underway. Moreover, we believe that it will be possible to find experimental conditions that would make it possible to obtain a microsphere with a narrower size distribution (which is under investigation in our group). Optical microscopy studies demonstrated that the average size of microspheres in a swollen state (microspheres are placed on a glass slide in the presence of water) is 5 µm. The scanning electron microscopy (SEM) image of the film formed from these microspheres is presented in Figure 3d. The SEM study showed that the microsphere size was significantly reduced upon drying. In this case, the structure of the microspheres changed; spherical objects disappeared, and a film with a developed surface structure was formed. The presence of a porous structure was confirmed by nitrogen isotherm adsorption–desorption measurements (Figure 3b). The study of the porous structure of crosslinked **PNaSS** microspheres demonstrated that the value of the specific surface area of the **PNaSS** microspheres was 1.55 m^2^/g (which is 1.5 times higher than that for microspheres with smooth surfaces). Differential curves of pore diameter distributions showed that the porous structure of **PNaSS** microspheres included mesopores with sizes ranging from 3 to 20 nm (Figure 3c). The appearance of mesopores was caused by the loose packing of polymer chains inside supramolecular structures. In the case of the microspheres coated with **PEDOT** film, the surface morphology of the deposited film was rougher than the film formed from pure microspheres. Due to the rough surface of the **PNaSS@PEDOT** microsphere film, the water contact angle increases (63° (Figure 3f), n = 5 as compared to the value of contact angle 35° for pure microsphere films (n = 5) and 50° for pure **PEDOT** film). For comparison, the image of surface of the pure **PEDOT** film is presented in Figure 3c.

The rough (porous) surface area is advantageous in testing the sensing properties of the **PEDOT** layer deposited onto microspheres; in this case, the total surface area is much higher than that of a planar **PEDOT** film. 

**Figure 4:**
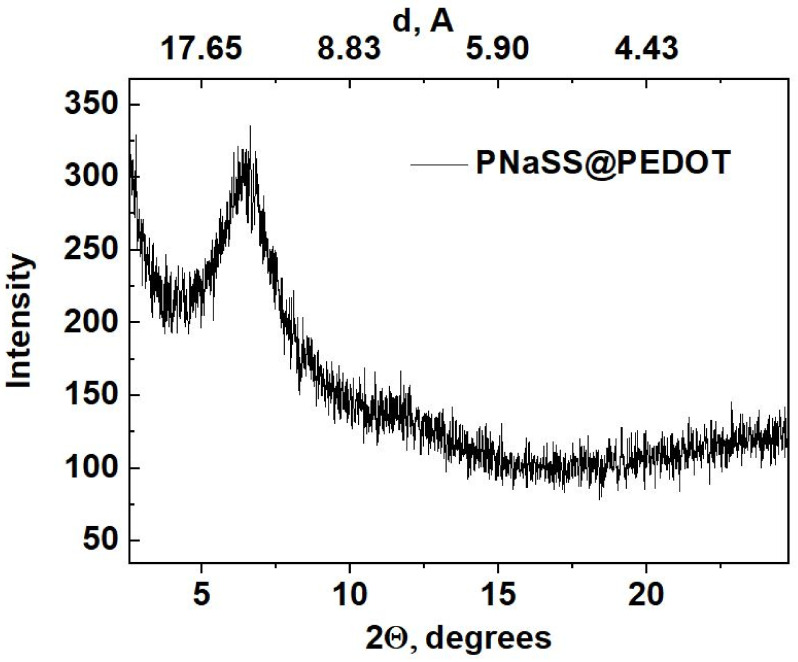
X-ray diffraction of **PNaSS@PEDOT** microsphere film deposited on FTO electrode.

To obtain more information about structure of the **PNaSS@PEDOT** microspheres films, the X-ray diffraction (XRD) measurements were performed (Figure 4).

The main peak (PEDOT (100)) is positioned at 2Θ = 6.55°, which corresponds to 13.48 Å; the peak width is 1.974° [44]. Based on this value, we estimated the size of the ordered domain using the Scherrer equation. The resulting value was 40.51 Å. 

Another less intense, but wide peak is observed at 2Θ = 12.83° (6.89 Å). Due to the low signal-to-noise ratio, it was impossible to calculate its width correctly.

### 3.3. Interaction of **PNaSS@PEDOT** Microspheres Films with E. coli

PEDOT polymer is composed of rigid planar aromatic repeat units [44,45,46]. Interactions between polymer chains (a combination of relatively weak van der Waals intermolecular associations, dipolar interactions, and electrostatic interaction, e.g., hydrogen bonds) facilitate electronic coupling between fragments, which is essential for charge transport. 

It is known that the oxidation state of **PEDOT** (the presence of reduced or oxidized monomer units) affects the interaction of bacteria with the surface (adhesion). Moreover, the nature of the surface onto which cells adhere, such as its softness or topography, affects the biological signaling pathways [1]. Generally, the physicochemical properties (pH, wettability, and roughness) of conducting polymers (e.g., PEDOT) can undergo reversible changes both in the bulk and at the surface as their electrochemical state is switched. Modulation of the physicochemical properties of the films provides an indirect tool for changing the adhesion behavior of cells.

Organic bio-electronic devices are commonly based on solution-cast thin films of PEDOT:PSS or similar semiconducting polymers [4]. In our work, we decided to use highly sulfonated microspheres as sulfo group-containing cores; it was supposed that the presence of these groups would improve, and stabilize the charge propagation from the sensing surface to the current collector electrode. In addition, the new original synthetic method for **PEDOT** allowed us to prepare a reduced **PEDOT** shell without by-products, which is desirable for applications in antifouling thin films. Moreover, the suspension of **PNaSS@PEDOT** microspheres is stable and can be deposited onto any surface with the formation of a thin film.

**Figure 5:**
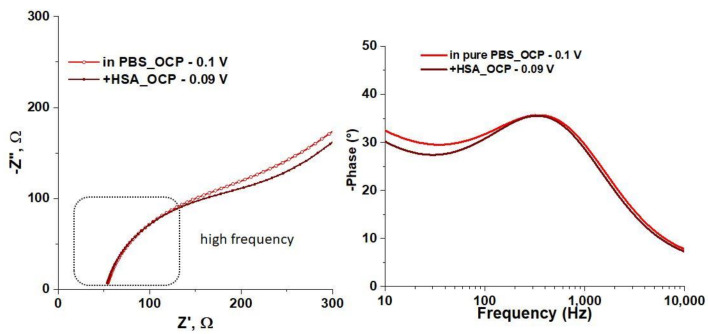
EIS of **PNaSS@PEDOT** microspheres film in the presence of human serum albumin (HSA) measured at different conditions: before and after applying HSA, (**a**) the Nyquist plots, and (**b**) the Bode plots.

In order to evaluate the antifouling properties of the **PNaSS@PEDOT** microsphere films, we decided to perform the tests involving human serum albumin (HSA), one of the most abundant plasma proteins. A solution of HAS was poured onto the horizontal film and left to stand for 30 min. Then, the EIS was measured in PBS solution at room temperature. For comparison, the **PNaSS@PEDOT** microsphere film before interaction with HSA was also measured in PBS; the results are presented in Figure 5.

Electrochemical impedance spectroscopy (EIS) measures the signal that changes after the adsorption of biomolecules from the solution on the electrode surface [1,2]. The signal is modified due to interfacial electron transfer between the solution species and the conducting electrode. The detected charge transfer resistance is caused by an increase in the number of target biomolecules bound to the working surface of the electrode [3,4,5,6]. Thus, the selectivity, sensitivity, time response, and detection limit of the method depend strongly on the properties of the material used in preparation of the working electrode. 

As shown in Figure 5, the Nyquist plots (Figure 5a) and Bode plots (Figure 5b) are similar before and after the application of HSA, particularly at high frequencies (represented by Z′), which is the most interesting frequency range for our purposes. A.F. Ogata et al. reported the detection of HSA with the help of virus-PEDOT film by measuring EIS in the concentration range from 50 nM to 5 µM; a monotonous increase in Z′ with increasing HSA concentration was observed [31]. Based on these results, it can be concluded that the presence of HSA does not affect the sensing ability of the **PNaSS@PEDOT** microsphere films. Similar results were observed for the zwitterionic PSBEDOT [41].

To obtain further information about the electrochemical performance of **PNaSS@PEDOT** microsphere films, the bacterial adhesion, antimicrobial, and releasing properties were studied using *E. coli* BL21 as a model species. 

In order to detect the adhesion of bacteria to the film, the following condition must be fulfilled: Rf << Rbac, where Rf is the resistance of the **PNaSS@PEDOT** microsphere film; Rbac is the ohmic resistance of the bacterial wall due to the tight junctional connection between the bacteria and the **PEDOT** film. The Rf value of the **PNaSS@PEDOT** microsphere film was determined from the EIS measurements (Figure 1) by fitting the measured data. The equivalent electrical circuit model consists of ohmic resistance of the solution (which is similar for all measurements), R = R_solution_ + R_f_. A similar circuit model was used by D.C. Martin et al. [18]. 

Naturally, the presence of the bacteria impedes the movement of ionic charges. The possibility of registering the electrical fingerprint of the bacterial wall depends on the relationship between the impedance attributed to the wall and the one attributed to the electrode. Larger electrodes capture the transmitted signal better, since their impedance values are lower than that of the bacterial wall. This phenomenon manifests itself in the appearance of the plateau at frequencies below 10 kHz for these electrodes. Similarly, regarding the impedance phase φ spectra, peaks are also observed. These peaks denote the capacitive contribution due to the presence of the bacterial wall.

**Figure 6:**
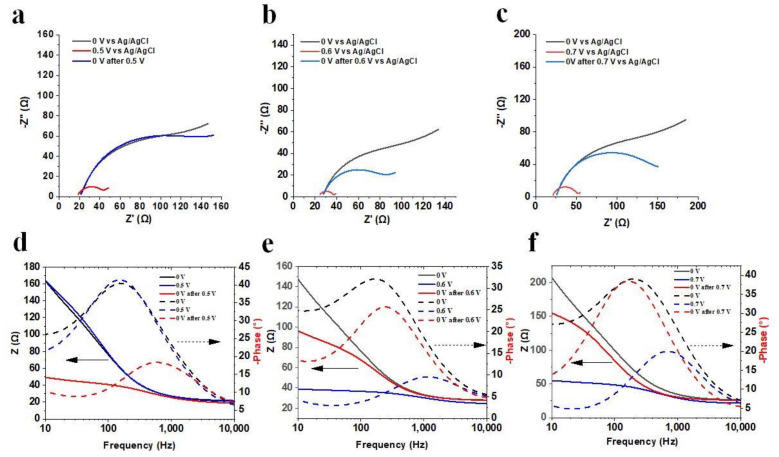
EIS in the form of Nyquist plots (**a**–**c**) and corresponding Bode plots (dashed line) with total impedance vs. frequency (solid line) (**d**–**f**) of **PNaSS@PEDOT** microspheres with *E. coli* at different applied potentials.

The **PNaSS@PEDOT** microsphere films were immersed in the solution containing 10^6^ colony-forming units (CFU)/mL of *E. coli* BL21 for 4 h; the procedure is described in detail in the experimental section. To prove that *E. coli* was present on the surface of the **PEDOT** film, energy-dispersive X-ray spectroscopy (EDX) was conducted, and the results are presented in Figure S5, SI. The EDX data for the **PEDOT** film with *E. coli* provides evidence of the bacteria’s presence (decreased weight fractions of S from 13.1 to 3.1 is observed). According to the weight fractions of the elements C, N and O can be considered to be major elements present in bacteria, and would be in a range from 80 to 90 percent [47]. On the other hand, the EDX data of the **PEDOT** film only has atoms related to its structure (Figure S5 top). Similar results from EDX data have been reported in the literature by Y.-J. Kim and co-workers [46]. The EIS of all **PNaSS@PEDOT** microsphere films with bacteria recorded at different applied potentials are presented in Figure 6. Firstly, the EIS was measured at the open circuit potential (0 V vs. Ag/AgCl reference electrode). Then, the oxidation potential (0.5 V, 0.6 V and 0.7 V vs. Ag/AgCl) was applied for 10 min in order to oxidize the **PEDOT** and to destroy the bacterial wall. Three different oxidation potentials were used to find the optimal value. Then, the EIS was measured at the original open circuit potential (0 V vs. Ag/AgCl), and the results were compared with the data from the first measurement (the open circuit potential, 0 V vs. Ag/AgCl). The corresponding Bode plots and the total impedance vs. frequency plots for each measurement are presented under the Nyquist plots (Figure 6d–f).

**Figure 7:**
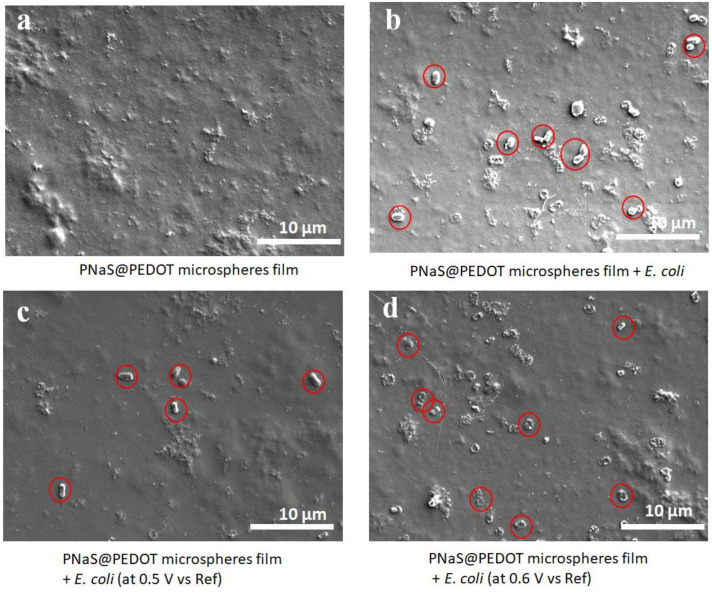
SEM images of **PNaSS@PEDOT** microsphere film (**a**), **PNaSS@PEDOT** microsphere film with *E. coli* (**b**), **PNaSS@PEDOT** microsphere film with *E. coli* oxidized at 0.5 V vs. Ag/AgCl (**c**), **PNaSS@PEDOT** microsphere film with *E. coli* oxidized at 0.6 V vs. Ag/AgCl (**d**).

The external modulation of the redox state of the **PNaSS@PEDOT** microsphere films controls the adhesion behavior and viability of the cells [46,47]. In our case, the **PEDOT** chains exist in the reduced state, which was confirmed by XPS spectra. The studies of bacterial attachment showed that the surface of **PNaSS@PEDOT** microsphere films was resistant to the attachment of *E. coli* at a high concentration (10^6^ CFU/mL), as demonstrated by the SEM images in Figure 7b. However, a small number of bacteria can be attached to the film surface, and these bacteria contribute to the changes observed in EIS data presented in Figure 6 as compared to the EIS of **PNaSS@PEDOT** microspheres film without *E. coli* (Figure 1). In the high frequency region, the radius of the semi-circle increases, which confirms the fact that the R_bac_ value is higher than the R_f_ of **PNaSS@PEDOT** microspheres.

In order to understand the effect of the oxidation state of **PNaSS@PEDOT** microspheres on the bacterial wall (possibility of the destruction of bacteria), the oxidation potentials at 0.5 V, 0.6 V, and 0.7 V vs. Ag/AgCl were applied. After applying oxidation potential to the **PNaSS@PEDOT** microsphere film with *E. coli* for 10 min, the potential was reduced to zero (open circuit potential). The SEM images of the **PNaSS@PEDOT** microsphere films after applying the oxidation potentials are presented in Figure 7c,d. It is seen that the number of attached bacteria was reduced after applying 0.5 V vs. Ag/AgCl potential, which is also confirmed by SEM and EIS. The Nyquist plots measured at 0 V before and after application of the oxidation potential are similar. It can be concluded that the oxidation potential of the 0.5 V vs. Ag/AgCl electrode is insufficient to destroy the bacterial wall. On the other hand, the oxidation potential of 0.6 V vs. Ag/AgCl (the SEM image is presented in Figure 7d) is suitable for the destruction of the bacterial wall. This finding is also confirmed by the EIS measurements shown in Figure 6b,e. The application of the high-oxidation potential of 0.7 V vs. Ag/AgCl electrode may lead to the partial rearrangement of the polymer chains (which has been discussed in our previous work) and partial distraction of **PNaSS@PEDOT** microsphere films (presented in SI Figure S6); thus, high oxidation potential is not appropriate [47]. Based on these data, it was concluded that the optimal oxidation potential is 0.6 V vs. Ag/AgCl reference electrode. 

To obtain additional proof of the fact that our **PNaSS@PEDOT** microsphere films (containing **PEDOT** synthesized in the reduced state) have the ability to diminish bacterial attachment, the reference experiment was conducted. The **PNaSS@PEDOT** microsphere film was oxidized at potential 0.5 V vs. Ag/AgCl and then immersed in the solution containing high amount of *E. coli* (10^6^ CFU/mL) for 4 h.

**Figure 8:**
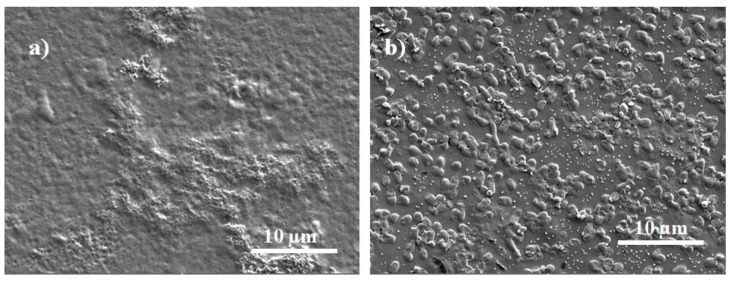
SEM images of the clean **PNaSS@PEDOT** microsphere film at potential 0.5 V vs. Ag/AgCl reference electrode (**a**) and with bacteria *E. coli* attached to the surface of the oxidized **PNaSS@PEDOT** microsphere film (**b**).

The results are presented in Figure 8. Almost the whole surface of the **PNaSS@PEDOT** microsphere film is covered with attached bacteria (Figure 8b), unlike the oxidized surface of **PNaSS@PEDOT** microsphere film without bacteria presented in Figure 8a.

The images given in Figure 8 are presented at higher magnifications in the SI (Figure S7).

In summary, we synthesized a new meta-material that can be used in manufacturing sensor layers. In the future, it is planned to test the wetting properties and antifouling properties using different methods [48,49]. Moreover, **PNaSS@PEDOT** microsphere film will be tested for the detection of other pathogenic strains.

## 4. Conclusions

In an exploration for semiconducting materials suitable for sensor applications, we have developed a new composite material based on **PEDOT** and microspheres (containing a sulfo group in each monomer unit). It has been demonstrated that innovative microspheres with a high concentration of the sulfur groups on their surface are coated by acid-assisted self-polymerization reaction with the formation of **PNaSS@PEDOT** microspheres as a stable suspension. The presence of sulfur groups in each monomer unit provides a conducting composite material even when **PEDOT** is in a reduced state. We have achieved a reproduceable **PNaSS@PEDOT** microsphere film suitable as an antifouling surface on the one hand, and as a sensor layer on the other. The antifouling properties were analyzed by testing with HAS. The sensing performance of the **PNaSS@PEDOT** microsphere film was achieved by applying electrochemical impedance spectroscopy on *E. coli* as a model species. The **PNaSS@PEDOT** microsphere film could detect the bacteria (*E. coli* B12 was used as a studying model) by applying electrochemical impedance spectroscopy (measured at open circuit potential) and the bacterial wall was successfully destroyed after applying an oxidation potential of 0.6 V vs. Ag/AgCl reference electrode. 

## Data Availability

Not applicable.

## Supplementary Materials

The following supporting information can be downloaded at: https://www.mdpi.com/article/10.3390/s22072784/s1: Preparation of **PNaSS@PEDOT** microspheres by inverse emulsion polymerization. Table S1: Inverse emulsion polymerization conditions of NaSS system and characteristics of obtained **PNaSS** polyelectrolyte microspheres; Figure S1: Optical microscopy of microspheres immediately after dialysis (dry on a glass slide); Figure S2: Various types of **PNaSS@PEDOT** microsphere deposition. Finding optimal method; Figure S3: Electrochemical impedance data for clean FTO electrode measured in PBS solution at OCP; Figure S4: Electrochemical impedance measurements of **PNaSS@PEDOT** microsphere films in the aqueous solutions of NaCl; Figure S5: Energy dispersive X-ray spectroscopy of **PEDOT** film (**top**) and **PEDOT** film with *E. coli* (**bottom**); Figure S6: SEM image of E. coli at **PNaSS@PEDOT** microspheres film oxidized at 0.7 V vs. Ag/AgCl reference electrode; Figure S7: SEM image of *E. coli* at oxidized **PNaSS@PEDOT** microsphere film.
