# Supplementary material for: Preparation of Smart Surfaces Based on PNaSS@PEDOT Microspheres: Testing of E. coli Detection
Source: Sensors (Basel). 2022 Apr 5;22(7):2784. doi: 10.3390/s22072784 (PMC9003540; doi:10.3390/s22072784)
Supplement: Supplementary file 1 [file sensors-22-02784-s001.zip › sensors-1648686-supplementary.pdf]

sensors

MDPI

Supporting Information

Preparation of Smart Surfaces Based on PNaSS@PEDOT Microspheres: Testing of *E. coli* Detection

Elena Tomšík [1,*], Svetlana Laishevkina [1,2], Jan Svoboda [1], Kristýna Gunar [1], Jiřina Hromádková [1] and Natalia Shevchenko [2,*]

1 Institute of Macromolecular Chemistry, Academy of Sciences of the Czech Republic, Heyrovsky Sq. 2, 162 06 Prague 6, Czech Republic; s.laishevkina@gmail.com (S.L.); svoboda@imc.cas.cz (J.S.); gunar@imc.cas.cz (K.G.); hromadkova@imc.cas.cz (J.H.)

2 Institute of Macromolecular Compounds, Russian Academy of Sciences, Bolshoy pr., 31, 199004 Saint-Petersburg, Russia

* Correspondence: tomsik@imc.cas.cz (E.T.); natali.shevchenko29@gmail.com (N.S.)

Preparation of PNaSS@PEDOT microspheres by inverse emulsion polymerization

Crosslinked poly(sodium styrene sulfonate) (PNaSS) microspheres were prepared by inverse emulsion polymerization (Table S1). The synthesis of PNaSS microspheres was carried out in a 100 mL flask equipped with a mechanical stirrer, a reflux condenser and a thermometer. 4-styrene sulfonic acid sodium salt NaSS (3 g), potassium persulfate $K_2S_2O_8$ (0.05 g, 2wt. % to Mn), N,N′-methylenebis(acrylamide) MBA (1.0 g, 30 wt.%) were successively dissolved in the aqueous phase using an ultrasound bath (Sapfir, Russia, 50 W generator). Tween 80 (0.3 g) was used as an emulsifier, it was previously dissolved in cyclohexane. * The composition of the reaction system H_2O:C_6H_{12} was 10:25 (ml). An aqueous solution of monomers was added to cyclohexane preheated to the reaction temperature under constant stirring. The reaction proceeded at a temperature of 70 °C and constant stirring (~ 500 rpm). The reaction proceeds for 3 hours. After the synthesis, cyclohexane was removed on a rotary evaporator at 45 °C. The crude polymer dispersion was purified by exhaustive dialysis (Orange Scientific; molecular weight cut-off = 12-14 kDa) for 3 days to remove the residual monomers and the surfactant. The dry residue (D.R.) was determined by gravimetric analysis: 1 mL of latex was dried to constant weight.

Table S1. Inverse emulsion polymerization conditions of NaSS system and characteristics of obtained PNaSS polyelectrolytes microspheres.

Sample	The composition of the reaction system				Characteristics of obtained polymer particles		
	$NaSS/H_2O$ (wt. %)	H_2O:C_6H_{12} (mL)	MBA/NaSS (wt. %)	Tween 80*/CDP (wt. %)	D** (μm)	S***. (m^2/g)	[SO_3^-] (**10^{-4}** mol/g)
PNaSS	20	**10:25**	**30**	1.6	1-5	1.55	1.54

Monomers: NaSS – sodium styrenesulfonate, MBA – N,N′-methylenebis(acrylamide). * The composition of the cyclohexane dispersion phase (CDP) was 1.6 wt.% of Tween 80. C6H12 – cyclohexane. ** Determined by optical microscopy. ***Nitrogen adsorption/desorption analysis was used to evaluate Brunauer-Emmett-Teller (BET) surface area (S).

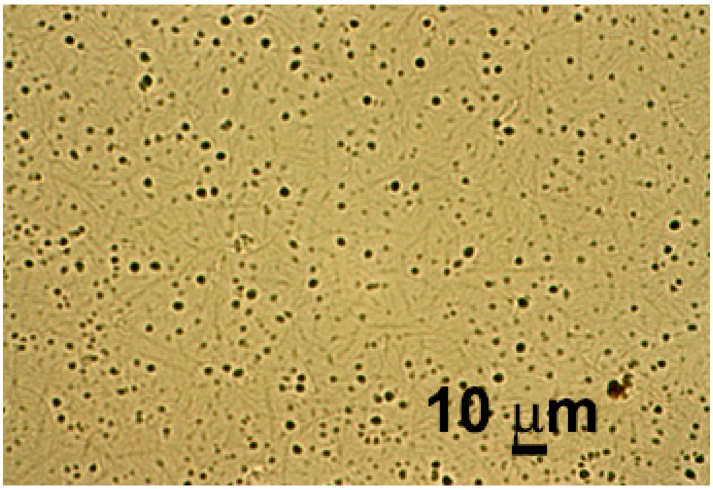

Figure S1. Optical microscopy of microspheres immediately after dialysis (dry on a glass slide).

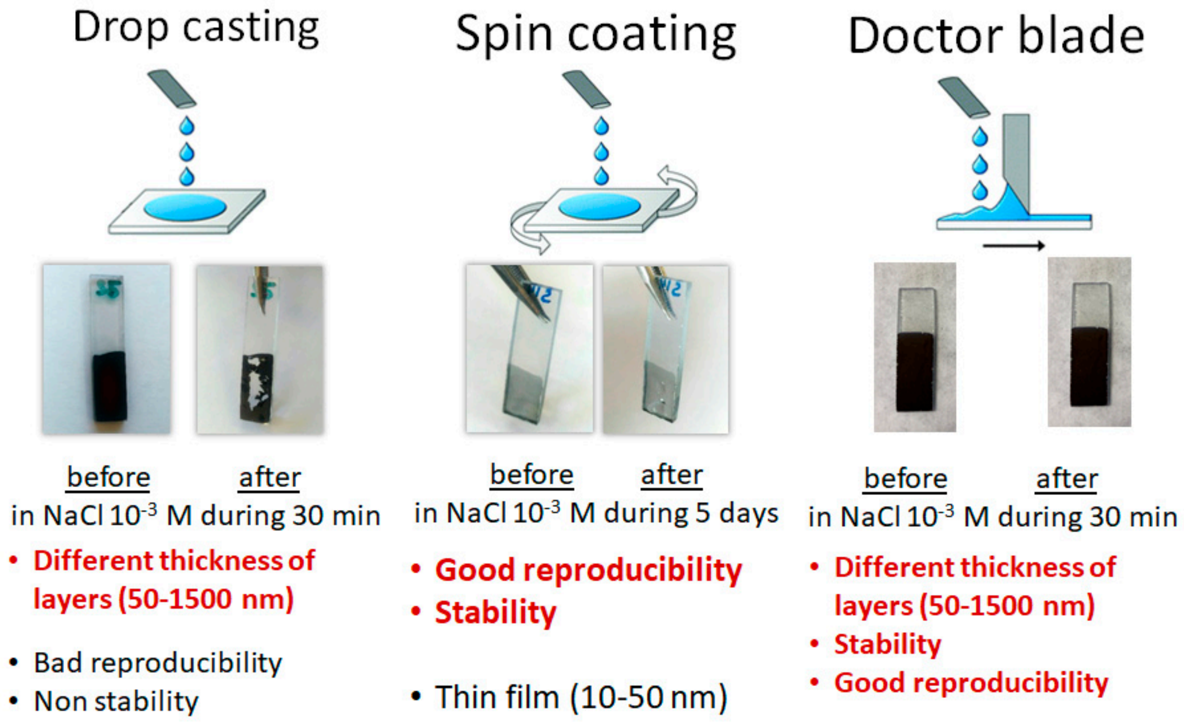

Figure S2. A various type of PNaSS@PEDOT microspheres deposition. Finding optimal method.

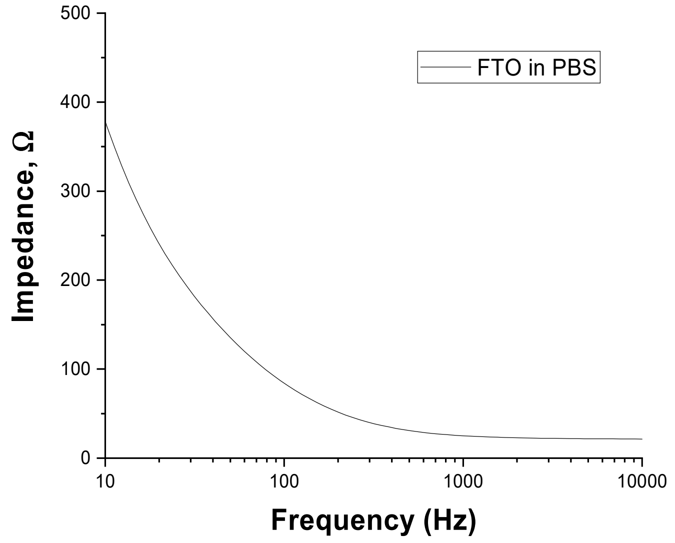

Figure S3. The Electrochemical impedance data for clean FTO electrode measured in PBS solution at OCP.

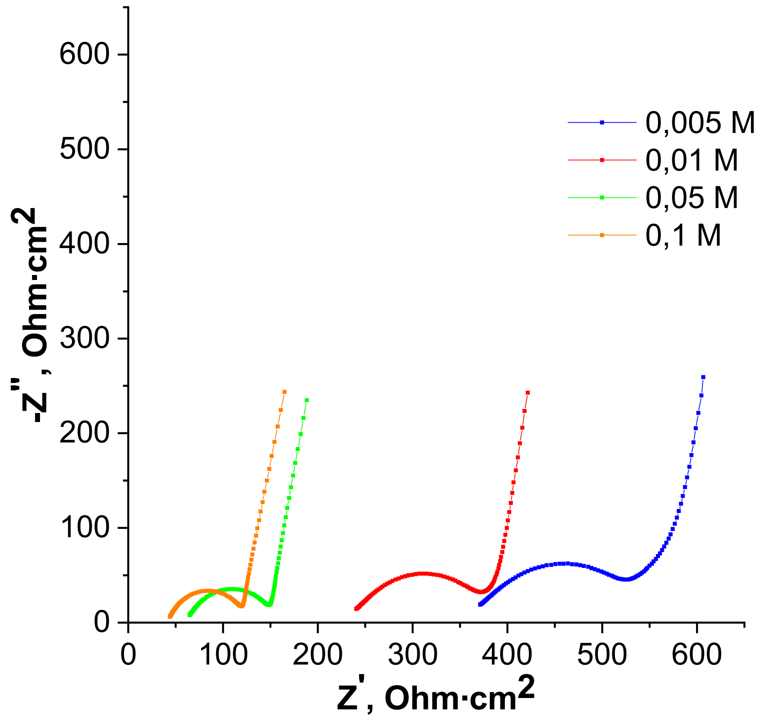

Figure S4. Electrochemical impedance measurements of PNaSS@PEDOT microspheres films in the aqueous solutions of NaCl.

The thickness of the layer is 1000 ±50 nm.

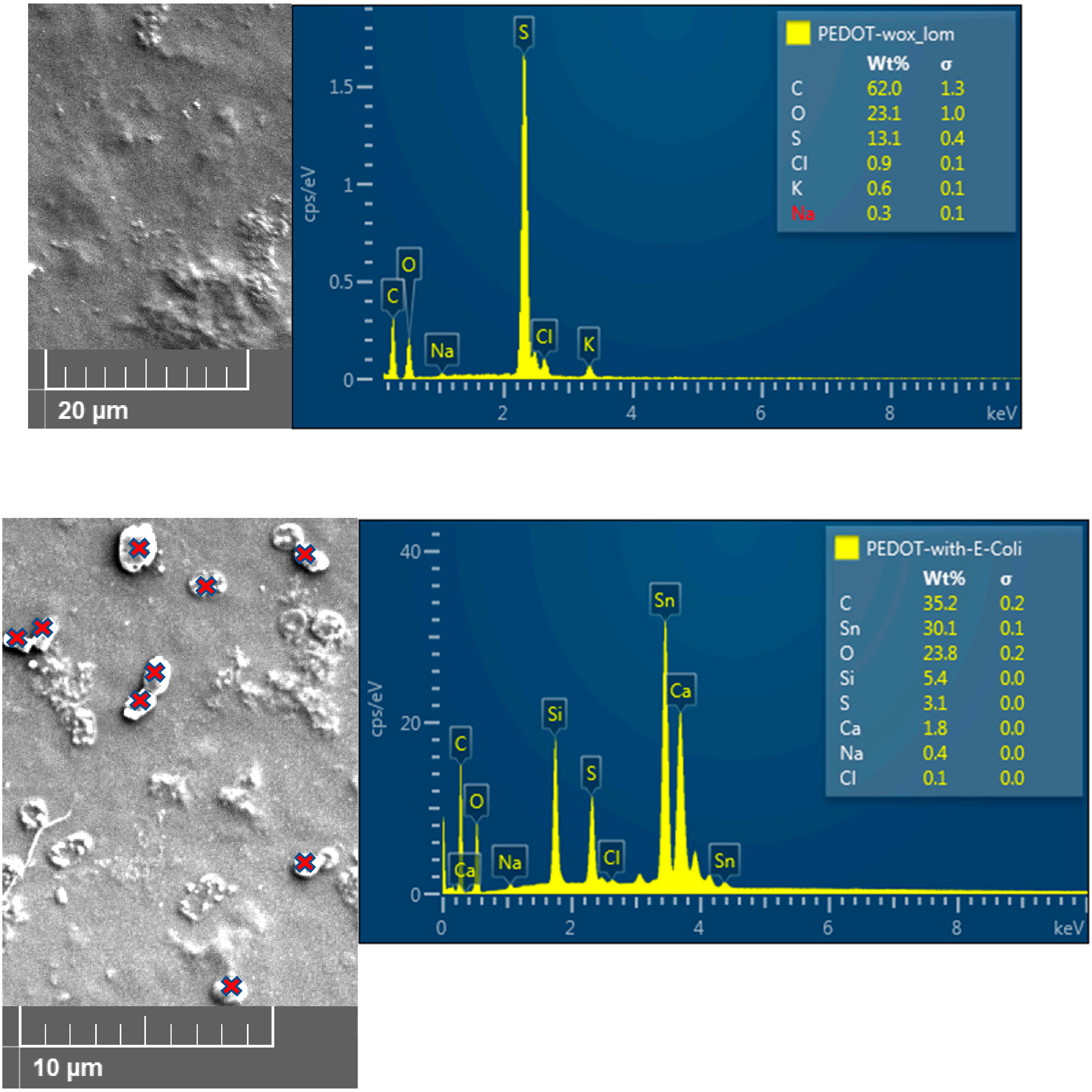

Figure S5. Energy dispersive X-ray spectroscopy of PEDOT film (top) and PEDOT film with *E. coli* (bottom).

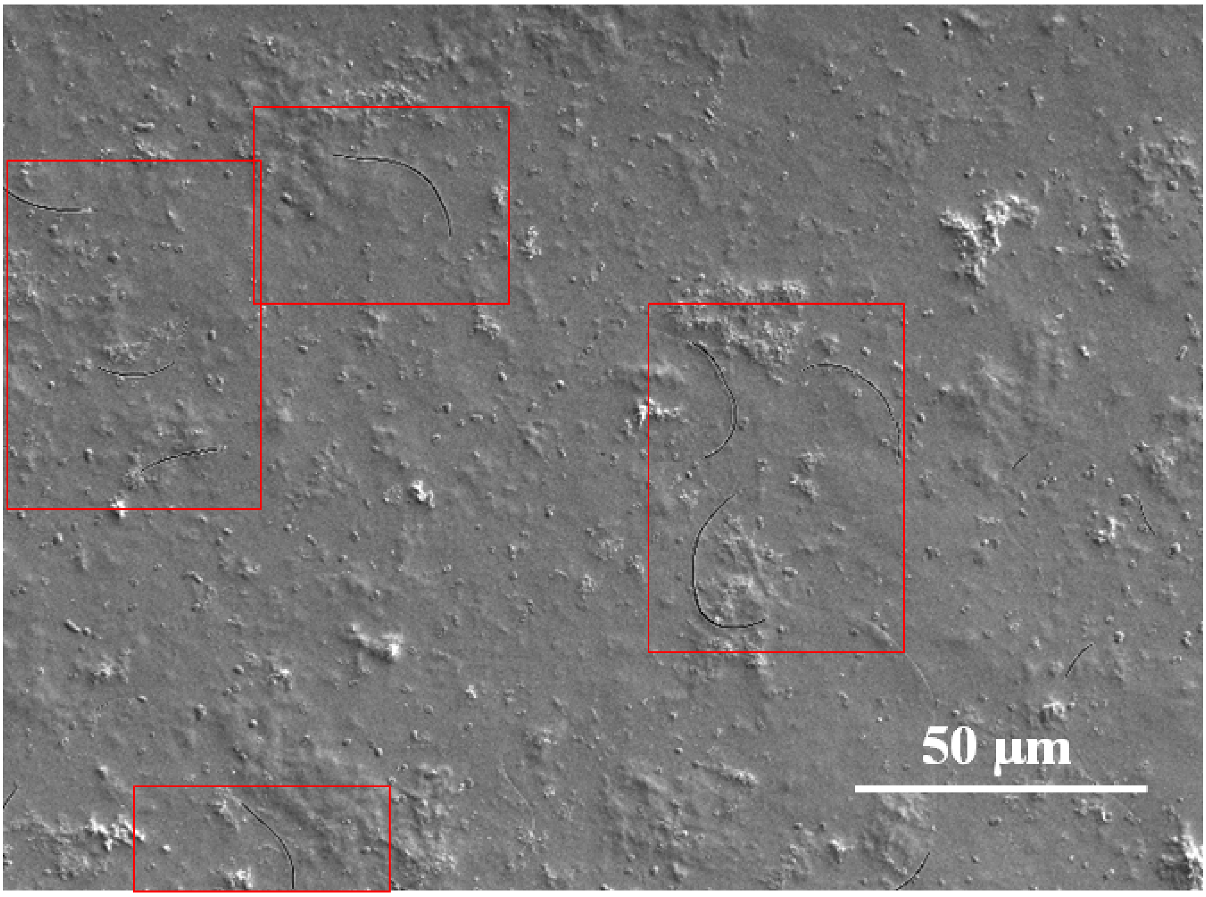

Figure S6. SEM image of *E. coli* at PNaSS@PEDOT microspheres film oxidized at 0.7 V vs Ag/AgCl reference electrode.

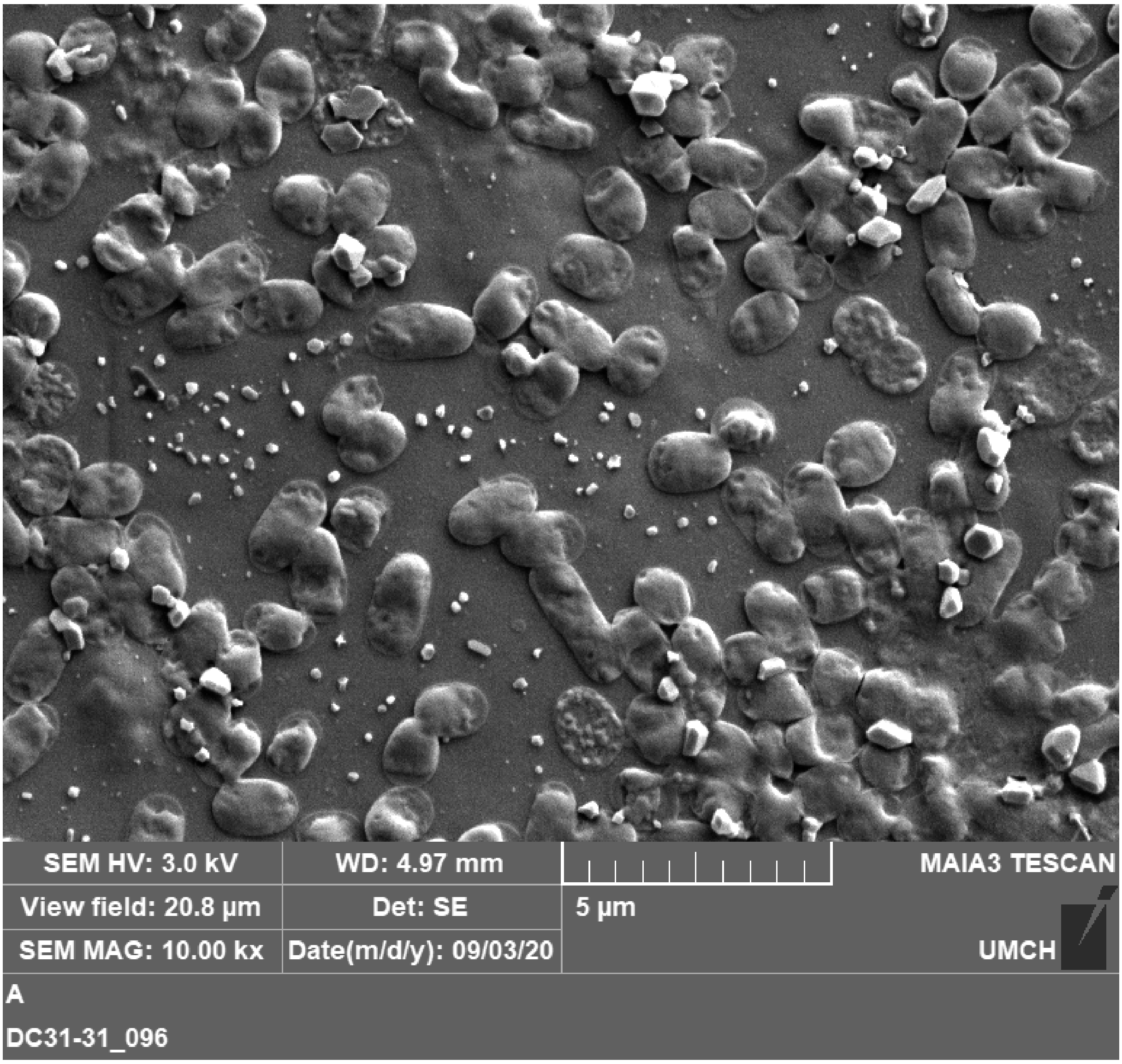

Figure S7. SEM image of *E. coli* at PNaSS@PEDOT microspheres film oxidized at 0.5 V vs Ag/AgCl reference electrode.
